# Ginsenoside Rb1 Improves Post-Cardiac Arrest Myocardial Stunning and Cerebral Outcomes by Regulating the Keap1/Nrf2 Pathway

**DOI:** 10.3390/ijms24055059

**Published:** 2023-03-06

**Authors:** Long Chen, Na Geng, Taiwei Chen, Qingqing Xiao, Hengyuan Zhang, Huanhuan Huo, Lisheng Jiang, Qin Shao, Ben He

**Affiliations:** 1 Department of Cardiology, Shanghai Chest Hospital, Shanghai Jiao Tong University School of Medicine, Shanghai 200030, China; 2 Department of Cardiology, Renji Hospital, Shanghai Jiao Tong University School of Medicine, Shanghai 200030, China

**Keywords:** cardiac arrest, myocardial stunning, ginsenoside Rb1, cardiopulmonary resuscitation, mitochondria, Nrf2

## Abstract

The prognosis of cardiac arrest (CA) is dismal despite the ongoing progress in cardiopulmonary resuscitation (CPR). ginsenoside Rb1 (Gn-Rb1) has been verified to be cardioprotective in cardiac remodeling and cardiac ischemia/reperfusion (I/R) injury, but its role is less known in CA. After 15 min of potassium chloride-induced CA, male C57BL/6 mice were resuscitated. Gn-Rb1 was blindly randomized to mice after 20 s of CPR. We assessed the cardiac systolic function before CA and 3 h after CPR. Mortality rates, neurological outcome, mitochondrial homeostasis, and the levels of oxidative stress were evaluated. We found that Gn-Rb1 improved the long-term survival during the post-resuscitation period but did not affect the ROSC rate. Further mechanistic investigations revealed that Gn-Rb1 ameliorated CA/CPR-induced mitochondrial destabilization and oxidative stress, partially via the activation of Keap1/Nrf2 axis. Gn-Rb1 improved the neurological outcome after resuscitation partially by balancing the oxidative stress and suppressing apoptosis. In sum, Gn-Rb1 protects against post-CA myocardial stunning and cerebral outcomes via the induction of the Nrf2 signaling pathway, which may offer a new insight into therapeutic strategies for CA.

## 1. Introduction

Sudden cardiac arrest (CA) carries a high burden of mortality and morbidity worldwide, despite the ongoing efforts to improve the “chain of survival” over the past 20 years [1]. A recent study showed that the global incidence of CA was around 3.7 million every year [2]. In America, the current survival rates of out-of-hospital CA are 11.4% and 10.4% for children and adults, respectively, contrasted with the rates for in-hospital CA, which are 41.1% and 25.8% for children and adults [3]. Of those survivors, up to 60% suffer from moderate to severe cognitive deficits, and 65% are attacked by post-arrest myocardial dysfunction, including left ventricular diastolic or systolic dysfunction, and low cardiac index [2]. In China, more than 500,000 new cases occur annually, and the prognosis, despite successful resuscitations and the return of spontaneous circulation (ROSC), is poor due to the limited options for treatment [4]. Paying attention to the pathogenesis of CA and searching for therapies that are more efficient and potent is thus, of the utmost importance.

CA results in whole-body ischemia reperfusion (I/R) injury, which is related to myocardial dysfunction and neurological deficit. Notably, mitochondria destabilization and oxidative stress act as core pathological components of CA and I/R injury [5,6]. Mitochondria is a substantial source of reactive oxygen species (ROS), and it also represents a target for its deleterious effects. Excessive oxidative stress may cause mitochondria destabilization, which induces the enhanced production of free radical, thus triggering a vicious cycle that aggravates oxidative injury, and thereby, affects oxidative phosphorylation and energy metabolism [7]. Therefore, targeting the mitochondria and oxidative stress may hold promise for therapeutic treatments.

Ginseng, a naturally occurring herb, has been widely used in East Asian countries such as China, Korea, and Japan for centuries to maintain body homeostasis and energy enhancement. Ginsenoside is the main bio-active component, which is extracted from ginseng. Currently, more than 100 ginsenosides have been identified [8], of which ginsenoside Rb1 (Gn-Rb1) is the most active and abundant monomer. A recent clinical study demonstrated the protective effect of Gn-Rb1 in chronic kidney disease, and the pharmacological mechanism involved anti-oxidative stress and anti-inflammation [9]. Previous works have observed the potential benefits of Gn-Rb1 in animal models of I/R settings for various organs including the heart [10,11,12], brain [13], spinal cord [14], intestine [15], and kidney [16]. Modern pharmacology researches have revealed multiple pharmacological properties of Gn-Rb1 on the cardiovascular system, including anti-oxidative, anti-apoptotic, and anti- inflammatory [10,11,12]. However, the effects of Gn-Rb1 against post-cardiac arrest syndrome, which is complicated by the whole-body I/R injury after ROSC following CA, has hitherto remained obscure.

In the current study, we found that the administration of Gn-Rb1 during the early cardio-pulmonary resuscitation (CPR) period improved post-CA myocardial stunning and secondary brain injury. These findings provided new insights into the role of Gn-Rb1 in cardioprotection, which could pave the way for developing novel therapeutic strategies for post-cardiac arrest syndrome.

## 2. Results

### 2.1. Baseline and Procedural Characteristics of the Animals

**Figure 1:**
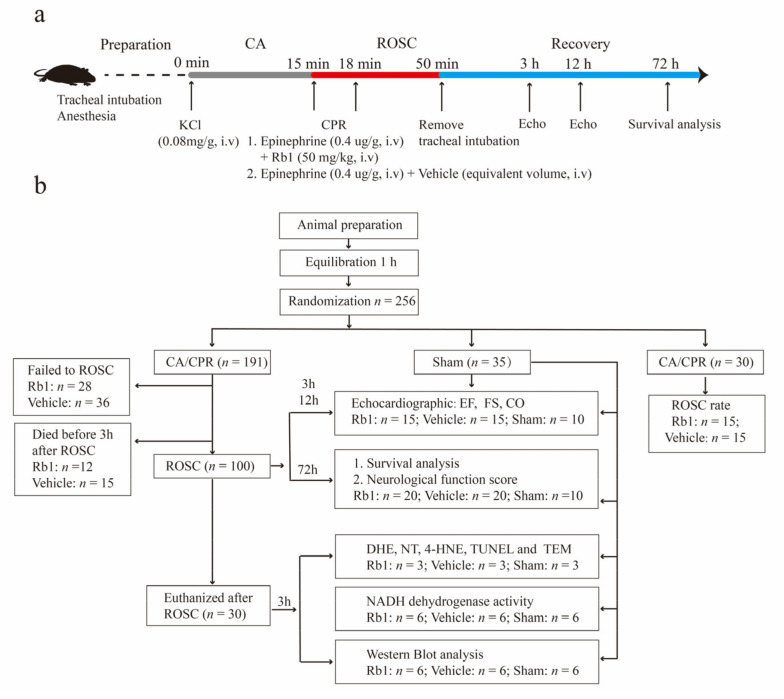
Flow chart of the experimental groups. (**a**) Schematic of the experimental workflow. min: minute; h: hour; min: minute; CA: cardiac arrest; CPR: cardiopulmonary resuscitation; KCl: potassium chloride; ROSC: return of spontaneous circulation; echo: echocardiography; Rb1: ginsenoside Rb1; (**b**) flow chart of the experimental groups. sham: mice underwent a sham surgery without CA/CPR; vehicle: mice subjected to CA/CPR without Rb1; *n*: number of mice; EF: ejection fraction; FS: fractional shortening; CO: cardiac output; DHE: dihydroethidium; 4-HNE: 4 hydroxynonenal; NT: nitrotyrosine; TUNEL: terminal dUTP nick-end labeling; NADH: NADH dehydrogenase; and TEM: transmission electron microscopy.

A total of 256 mice were subjected to the sham operation (*n* = 35) or the potassium chloride-induced CA/CPR (*n* = 221). In the CA/CPR group, 30 mice were used to summarize ROSC-related characteristics. Of the other 127 successfully resuscitated mice, 100 survived for more than 3 h. The remaining 100 resuscitated mice were then randomly assigned to either the 72 h group (*n* = 40) or the 3 h group (*n* = 60) for the following investigation (Figure 1b). There were no differences in the resuscitation-related variables between the post-arrest mice treated with Gn-Rb1 or not, such as chest compression rate, ventilator parameters, body weight, heart rate, body temperature, and so on.

### 2.2. Gn-Rb1 Treatment Improved the Prognosis of CA/CPR Mice

**Figure 2:**
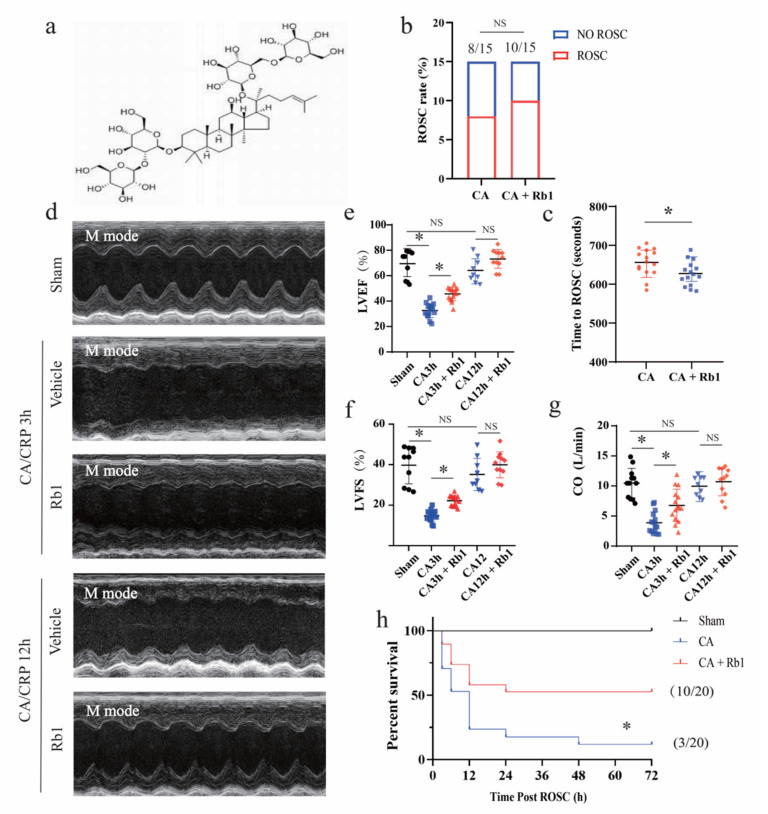
Gn-Rb1 treatment attenuated myocardial dysfunction and improved survival rate after CA/CPR. (**a**) Structure of ginsenoside Rb1. (**b**) Return of spontaneous circulation (ROSC) rate (*n* = 15 per group). (**c**) Time for ROSC (*n* = 15 per group). (**d**–**g**) Cardiac dysfunction measured by echocardiography (*n* = 9–15 per group). (**h**) Kaplan–Meier curve demonstrating survival following CA/CPR (*n* = 10 in sham, *n* = 20 in CA, and CA + Rb1 group). * *p* < 0.05; and NS = not significant. LVEF: left ventricular ejection fraction; LVFS: left ventricular fractional shortening; CO: cardiac output; CA: cardiac arrest; and Rb1: ginsenoside Rb1; h: hour.

Gn-Rb1 has been shown to protect the heart against I/R injury or ameliorate myocardial dysfunction in a different context [17,18,19,20,21]. However, the role of Gn-Rb1 in CA/CPR remains unknown. In the CA/CPR mouse model, no difference was observed in the ROSC rate between the CA group and CA+Rb1 group (53.3% vs. 66.7% and *p* = 0.248), whereas the time for ROSC was significantly improved in the Gn-Rb1 treated group (*p* < 0.05) (Figure 2b,c). The effect of Gn-Rb1 in impro ving the ROSC time suggested that Gn-Rb1 may be involved in the CA/CPR period.

The effect of Gn-Rb1 on the post-resuscitation restoration of cardiac function was ascertained by transthoracic echocardiography. As shown in Figure 2d–g, CA/CPR induced severe depression of the LVEF, LVFS and CO during the first 3 h following the ROSC, which were significantly improved by the Gn-Rb1 treatment (32.65 ± 1.42% vs. 45.70 ± 1.36%, *p* < 0.05; 14.78 ± 2.78% vs. 22.01 ± 3.08%, *p* < 0.05, and 3.88 ± 0.46 vs. 6.76 ± 0.70, *p* < 0.05, respectively). Of note, the LVEF, LVFS, and CO did not differ significantly between the sham-operated mice and the 12 h post-arrest mice, regardless of treatment with Gn-Rb1 or not. The survival of mice in the CA group and CA+Rb1 group was monitored for 72 h (15%, 3 of 20 vs. 50%, 10 of 20, and *p* < 0.05, respectively). All ten mice in the sham group survived. The Kaplan–Meier survival curves indicated a rapid decline in survival within the first 12 h after ROSC (Figure 2h). Overall, these results indicate that Gn-Rb1 treatment during the early stage of CPR preserved the cardiac function and improved survival in CA/CPR mice.

### 2.3. Gn-Rb1 Attenuated Myocardial Oxidative Stress Following CA/CPR

**Figure 3:**
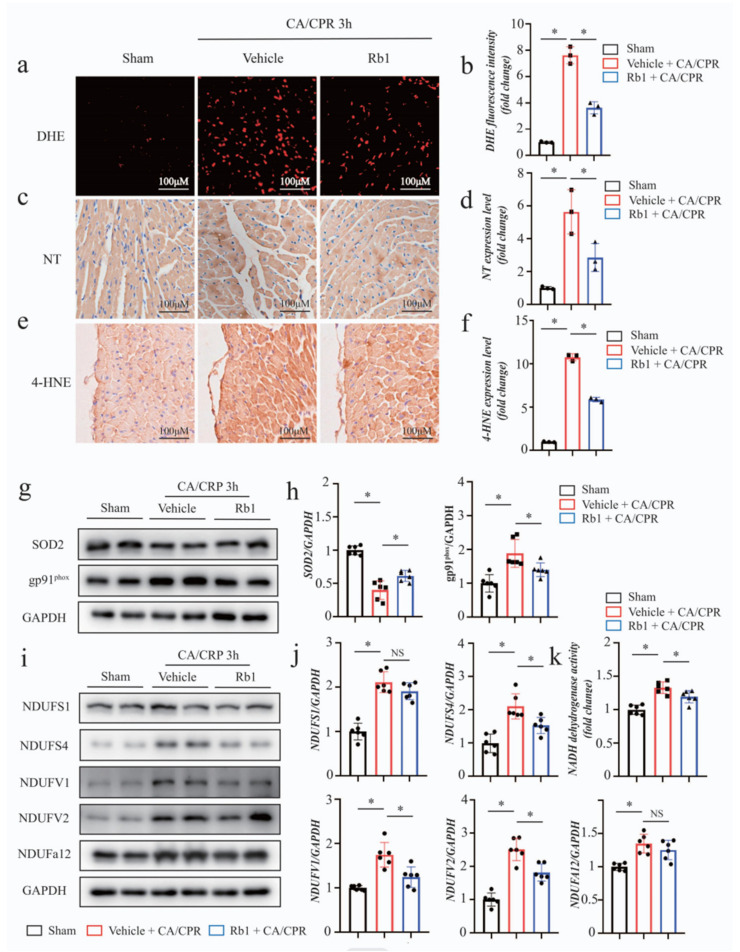
Gn-Rb1 attenuated oxidative stress in the heart following CA/CPR. (**a**,**b**) Representative immunofluorescence staining images and quantitative result of dihydroethidium (DHE), performed to assess myocardial ROS accumulation (*n* = 3 per group). (**c**,**d**) Representative immunohistochemical staining images and quantitative result of nitrotyrosine (NT), performed to assess myocardial nitrotyrosine production in different groups (*n* = 3 per group). (**e**,**f**) Representative immunohistochemical staining images and quantitative result of 4 hydroxynonenal (4-HNE), performed to assess myocardial lipid peroxidation (*n* = 3 per group). (**g**,**h**) Western blot analysis of antioxidant proteins SOD_2_ and oxidative markers gp91^phox^ in different cardiac homogenates (*n* = 6 per group). (**i**,**j**) Western blot analysis of subunits of NADH dehydrogenase in different cardiac homogenates (*n* = 6 per group). (**k**) NADH dehydrogenase was assessed using an ELISA assay 3 h following CA/CPR (*n* = 6 per group). CA: cardiac arrest; CPR: cardiopulmonary resuscitation; Rb1: ginsenoside Rb1. * *p* < 0.05; and NS = not significant.

CA/CPR is known to trigger oxidative damage, which contributes to myocardial dysfunction [22]. To probe the mechanisms underlying the cardioprotection of Gn-Rb1, we examined the effects of Gn-Rb1 in regulating CA/CPR-induced myocardial oxidative stress. As shown in Figure 3a–f, CA/CPR remarkably induced the production of superoxide accumulation, as shown by DHE staining, as well as peroxide byproducts, such as 4 hydroxynonenal (4-HNE) and nitrotyrosine (NT). As predicted, Gn-Rb1 improved the deposition of CA/CPR-induced ROS in the myocardium. In addition, the changes in the antioxidant proteins SOD_2_ and oxidative markers gp91 were a partial remission by Gn-Rb1 (Figure 3g,h).

As a potential site to drive the ROS production, NADH dehydrogenase was activated in cardiomyocytes during reperfusion [23]. Thus, we wondered what changes in NADH dehydrogenase would occur after CA/CPR. Our results indicated that CA/CPR activated NADH dehydrogenase, while Gn-Rb1 reduced its activity. In accordance, Gn-Rb1 inhibited the protein expression of some subunits of NADH dehydrogenase, such as NDUFS4, NDUFV1, and NDUFV2 (Figure 3i–k).

Collectively, these data indicated that the improvement of cardiac dysfunction by Gn-Rb1 is partly due to its antioxidant properties.

### 2.4. Gn-Rb1 Improves Mitochondrial Homeostasis and Energy Metabolism following CA/CPR

Multiple mechanisms underlying myocardial stunning have been reported, among which metabolic destabilization is one of the main culprits [24]. Mitochondrial homeostasis is a key mechanism contributing to energy metabolism. We therefore assessed the role of Gn-Rb1 on the mitochondrial dynamics and morphology, as well as ATP production, in the CA/CPR mice model.

**Figure 4:**
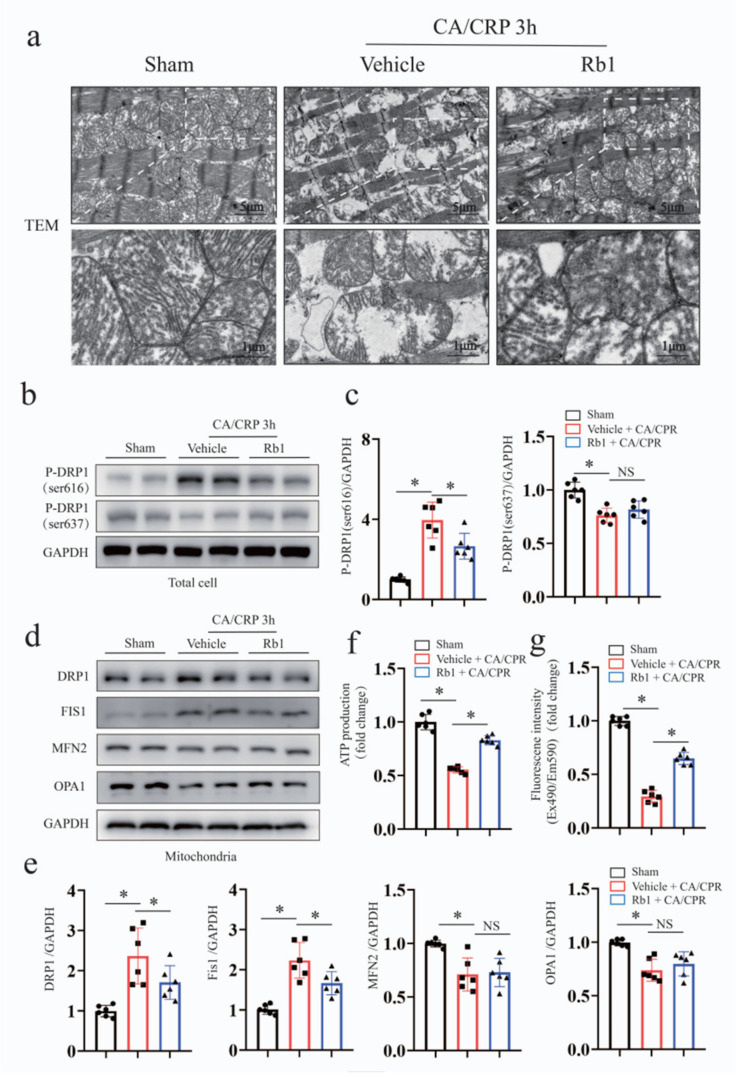
Gn-Rb1 improves mitochondrial homeostasis and energy metabolism in the heart following CA/CPR. (**a**) Transmission electron microscopy was performed to observe the ultrastructure of post-CA myocardial tissues (*n* = 3 per group). (**b**,**c**) Representative Western blots and quantitative analysis of total p-Drp1 (Ser 616) and p-Drp1 (Ser 637), as well as GAPDH protein expression (*n* = 6 per group). (**d**,**e**) Representative Western blots and quantitative analysis of mitochondrial Drp1, Fis1, Mfn2, Opa1, and VDAC1 protein expression (*n* = 6 per group). (**f**) The total ATP levels in different group were determined by Luciferase assay (*n* = 6 per group). (**g**) The membrane potential in isolated mitochondria was assessed by Luciferase assay (*n* = 6 per group). CA: cardiac arrest; CPR: cardiopulmonary resuscitation; Rb1: ginsenoside Rb1. * *p* < 0.05; and NS = not significant.

As shown in Figure 4a, the TEM analysis of the myocardial mitochondrial in CA mice revealed a substantial loss of matrix density, swelling, and cristae disruption. However, those pathological abnormalities were partially reversed by Gn-Rb1. Mitochondrial fusion and fission are considered the basic mechanisms for maintaining mitochondrial dynamics and morphology. As shown in Figure 4b,e, CA/CPR induced Drp1 translocation to the mitochondria and triggered phosphorylation of Drp1 at serine 616, whereas the Gn-Rb1 treatment prevented this effect. Notably, no significant differences were observed in the mitochondrial fusion proteins, such as OPA1 and MFN2. A reduction in the mitochondrial membrane potential (△ψ) and ATP production are hallmarks of mitochondrial dysfunction, shown in Figure 4f,g. However, Gn-Rb1 treatment improved the pathological status.

### 2.5. Gn-Rb1 Activates the Keap1/Nrf2 Signaling Pathway

**Figure 5:**
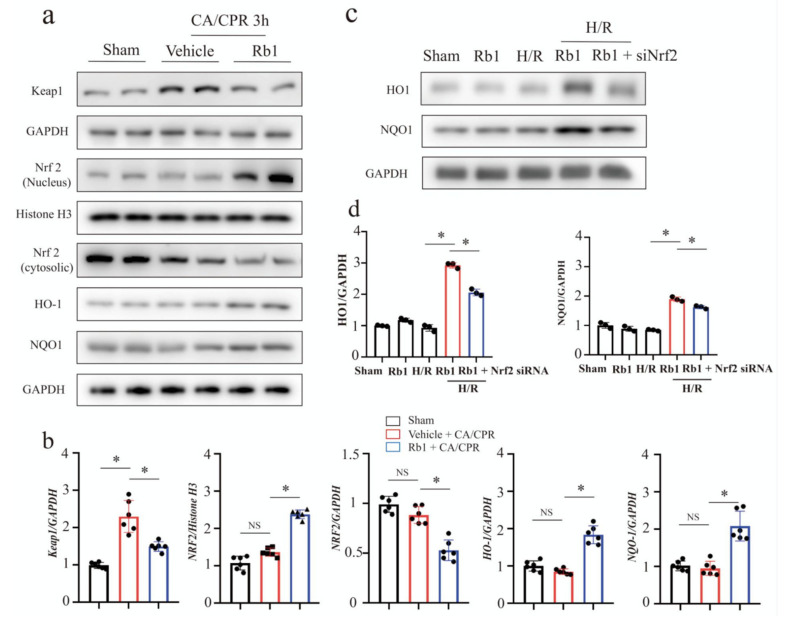
Gn-Rb1 activates the Keap1/Nrf2 signaling pathway in the heart following CA/CPR. (**a**,**b**) Representative Western blots and quantitative analysis of Keap1, nuclear and cytosolic Nrf2, HO-1, and NQO1 in the myocardium for 3 h, following CA/CPR in different groups (*n* = 6). (**c**,**d**) NRCMs were transfected with small interfering RNA targeting Nrf2 and exposed to hypoxia for 12 h, and then treated as indicated during reoxygenation for 3 h. Representative Western blots and quantitative analysis of HO-1 and NQO1 were assessed (*n* = 3). H/R: hypoxia/reoxygenation; CA: cardiac arrest; CPR: cardiopulmonary resuscitation; and Rb1: ginsenoside Rb1. * *p* < 0.05; and NS = not significant.

Next, we investigated the molecular mechanisms of Gn-Rb1 protecting the myocardium. The Keap1/Nrf2 axis is the key for the cellular regulation of redox homeostasis, mitochondrial physiology, and metabolism [20,25]. Upon cardiac I/R injury (I/R), Keap1 is inactivated and NRF2 accumulates in the nucleus. Activation of Nrf2 attenuates myocardial I/R injury [26,27]. Studies have indicated that Gn-Rb1 may participate in the regulation of the Nrf2 signaling pathway to counteract I/R injury and oxidative damage [28,29]. Accordingly, we assumed that Gn-Rb1 attenuated oxidative stress and improved mitochondrial homeostasis, partly empowered by the activation of the Nrf2 signaling pathway. As shown in Figure 5a,b, Gn-Rb1 attenuated the CA/CPR-induced up-regulation of keap1, a main repressor of the Nrf2 signal. In parallel, Gn-Rb1 promoted the translocation of Nrf2 into the nucleus, while the level of Nrf2 in the cytoplasm was down-regulated accordingly. There was no difference in the HO-1 and NQO1 proteins and the Nrf2 downstream antioxidant genes in the CA group as compared to sham, while all of which were partly up-regulated by the Gn-Rb1 treatment. This detailed mechanism needs to be further explored. Next, we introduced siRNA to knock down the Nrf2 expression (Figure 5c,d). Of note, the Nrf2 knockdown partly attenuated the Gn-Rb1-induced HO-1 expression and NQO1 expression in the context of hypoxia/reoxygenation (H/R). Collectively, the activation of the Keap1/Nrf2 axis may, in part, explain the protective effect of Gn-Rb1 in CA/CPR-induced myocardial injury.

### 2.6. Gene Knockdown of Nrf2 Attenuates the Ameliorative Effect of Gn-Rb1 on Oxidative Stress after Hypoxia/Reoxygenation(H/R)

**Figure 6:**
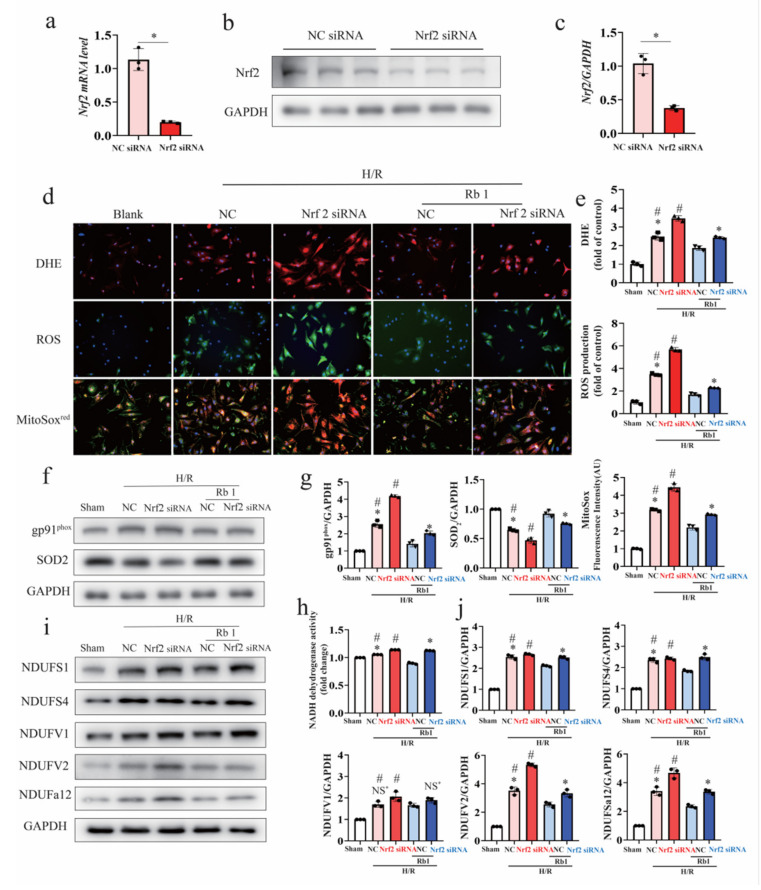
Gene knockdown of Nrf2 attenuates the ameliorative effect of ginsenoside Rb1 on oxidative stress after H/R. (**a**–**c**) The mRNA and protein expression of Nrf2 were assessed 48 h after Nrf2 siRNA or NC siRNA transfection in NRCM. (**d**,**e**) Representative fluorescent images of DHE, DCFH-DA, and MitoSOX red in the presence of Nrf2 siRNA or NC siRNA with or without Gn-Rb1 treatment (10 μM) in NRCM after H/R insult (*n* = 3). (**f**,**g**) Western blot analysis of antioxidant proteins SOD_2_ and oxidative markers gp91 in different groups. (**h**) NADH dehydrogenase activity was assessed using an ELISA assay in different groups (*n* = 3 per group). (**i**,**j**) Western blot analysis of subunits of NADH dehydrogenase in different groups (*n* = 3 per group). H/R: hypoxia/reoxygenation; DHE: dihydroethidium; ROS: Reactive oxygen species; and Rb1: ginsenoside Rb1. # *p* < 0.05 vs. Sham; * *p* < 0.05 vs. NC+Rb1+H/R; and NS = not significant.

To further substantiate the involvement of Nrf2 signaling in the protective action of Gn-Rb1 in the mouse CA/CPR model, NRCM were transfected with NC siRNA or Nrf2 siRNA. As shown in Figure 6a–c, the siRNA transfection decreased Nrf2 mRNA expression by ≥70% in NRCM. The intracellular ROS level was determined by DHE staining and a fluorescent probe (DCFH-DA). Superoxide within the mitochondrial was analyzed using the MitoSOX^red^ reagent. Gn-Rb1 alleviated H/R-induced intracellular oxidative stress and mitochondrial ROS production, while the gene knockdown of Nrf2 partly abrogated the antioxidant effects (Figure 6d,e). Consistent with this, changes in gp91 and SOD_2_ protein expression were affected by the knockdown of Nrf2 (Figure 6f,g). In addition, we examined the protein levels of the subunits of NADH dehydrogenase. Among the five subunits, NDUFV1 was not affected by the Nrf2. However, the Nrf2 knockdown partly offset the effect of Gn-Rb1 on the NADH dehydrogenase activity (Figure 6h,i).

### 2.7. Gene Knockdown of Nrf2 Attenuates the Ameliorative Effect of Gn-Rb1 on Mitochondrial Injury and Metabolic Destabilization after Hypoxia/Reoxygenation

**Figure 7:**
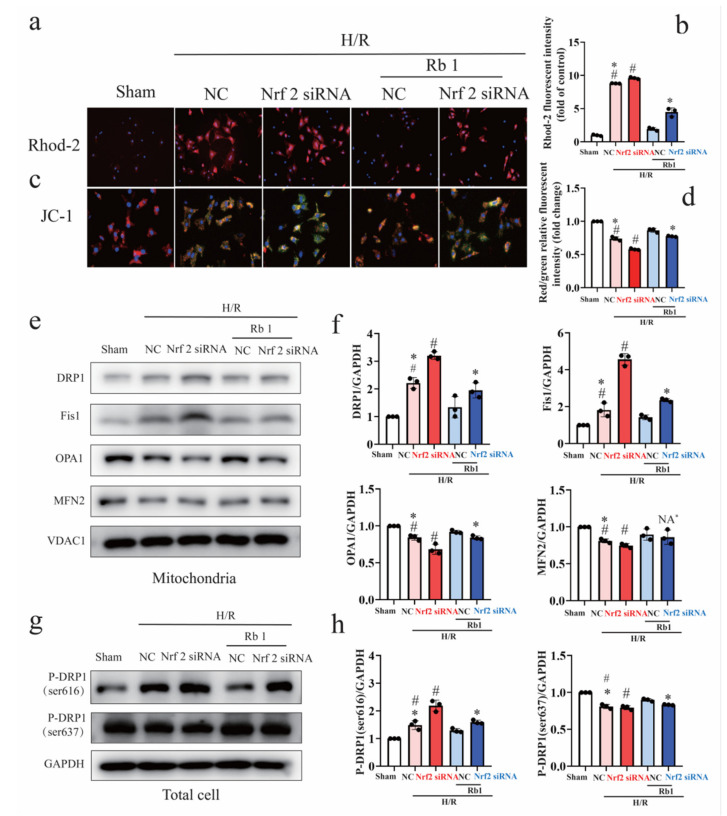
Gene knockdown of Nrf2 attenuates the ameliorative effect of ginsenoside Rb1 on mitochondrial injury and metabolic destabilization after H/R. (**a**–**d**) Representative fluorescent images of Rhod-2 and JC-1 in the presence of Nrf2 siRNA or NC siRNA with or without Rb1 treatment (10 μM) in NRCMs after H/R insult (*n* = 3). (**e**,**f**) Representative Western blots and quantitative analysis of mitochondrial Drp1, Fis1, Mfn2, Opa1, and VDAC1 protein expression (*n* = 3). (**g**,**h**) Representative Western blots and quantitative analysis of total p-Drp1 (Ser 616), p-Drp1 (Ser 637), and GAPDH protein expression (*n* = 3). H/R: hypoxia/reoxygenation; Rb1: ginsenoside Rb1. # *p* < 0.05 vs. Sham; * *p* < 0.05 vs. NC+Rb1+H/R.

Mitochondrial calcium overloading and the drop of the mitochondrial membrane potential (△Ψm) were hallmarks of mitochondrial dysfunction. Therefore, we measured mitochondrial Ca^2+^ with Rhodamine-2 (Rhod-2) and measured mitochondrial membrane potential with JC-1 staining. As shown in Figure 7a–d, the enhancement of mitochondrial Rhod-2 fluorescence and reduction in mitochondrial transmembrane potential after reoxygenation were improved partially by Gn-Rb1, while the gene knockdown of Nrf2 partly abrogated the protective effects. We next examined the impact of Nrf2 on mitochondrial morphology. Notably, Nrf2 regulated mitochondrial fission proteins, such as p-Drp1 (ser616) in total cell and Drp1 or Fis1 in mitochondrial, while it did not affect mitochondrial fusion proteins such as MFN2 and OPA1 (Figure 7e–h).

### 2.8. Gn-Rb1 Treatment Improved Neurological Outcomes

**Figure 8:**
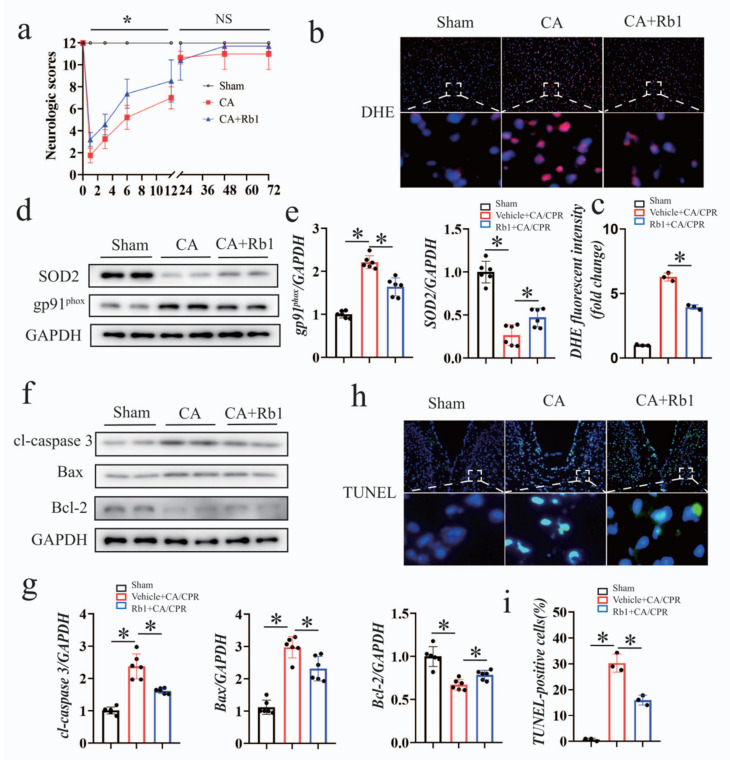
Gn-Rb1 treatment improved neurological outcomes after CA/CPR. (**a**) The neurological function score was assessed in the surviving mice within 72 h at indicated time points after CA/CPR. (**b**,**c**) Representative immunofluorescence staining images of dihydroethidium (DHE) performed to assess brain tissue ROS accumulation (*n* = 3 per group). (**d**,**e**) Western blot analysis of antioxidant proteins SOD_2_ and oxidative markers gp91 in different groups (*n* = 6 per group). (**f**,**g**) Representative immunofluorescence staining images of TUNEL performed to assess the number of TUNEL-positive cells in the brain tissue (*n* = 3 per group). (**h**,**i**) Representative Western blots and quantitative analysis of Bax, Bcl-2, and cleaved caspase-3 in different groups (*n* = 6 per group). DHE: dihydroethidium; CA: cardiac arrest; CPR: cardiopulmonary resuscitation; and Rb1: ginsenoside Rb1. * *p* < 0.05; and NS = not significant.

Neurological damage is one of the major cause of disability and death after CA/CPR. As shown in Figure 8a, the neurological deficit scores were dramatically improved in the Gn-Rb1-treated group before 24 h following CA/CPR. However, the differences between the two groups were not statistically significant at 72 h. Gn-Rb1 improved CA/CPR-induced oxidative injury and cell apoptosis, as shown in DHE staining and TUNEL staining. The changes in the antioxidant proteins SOD_2_ and oxidative markers gp91, as well as apoptosis-related or anti-apoptosis-related proteins also support this point of view (Figure 8b–i).

## 3. Discussion

The present investigations offered the following new insights concerning the effects of Gn-Rb1 in post-CA myocardial stunning: (a) Gn-Rb1 significantly improved long-term survival during the post-resuscitation period, but did not affect the ROSC rate. (b) Gn-Rb1 ameliorated CA/CPR-induced mitochondrial destabilization and oxidative stress partially via the activation of Keap1/Nrf2 axis. (c) Gn-Rb1 improved neurological outcome after resuscitation, partially by balancing the oxidative stress and suppressing apoptosis. In summary, these findings provided the first evidence that Gn-Rb1 protected against CA-induced myocardial stunning through ameliorating mitochondrial destabilization and oxidative stress in a Nrf2-dependent manner. Our findings provide a valuable reference and great insight for developing new agents to treat CA.

Previous researchers have suggested that post-CA myocardial dysfunction was reversible, and that the evolving process was consistent with myocardial stunning [30,31]. This is in agreement with clinical observations and our findings, to a certain extent. After a 12 h recovery period, the cardiac systolic and diastolic function largely returned to normal levels (Figure 2). Therefore, the effect of Gn-Rb1 on cardiac function, at this time point, was slightly less pronounced. However, insufficient cardiac output in the early post-resuscitation phase may worsen global I/R injuries, and contributes to the early deaths [32]. Accordingly, early improvement of myocardial stunning and increased support for the circulatory system are critical. We found that Gn-Rb1 significantly improved the cardiac contractility, output, and diastolic functions 3 h after the ROSC, compared with the vehicle group (Figure 2). Actually, an abundance evidence has supported the notion that Gn-Rb1 could improve cardiac function and remodeling in decompensated heart failure [19,20,33]. However, the role of Gn-Rb1 in post-cardiac arrest syndrome has not yet been identified. We demonstrated that Gn-Rb1 induced positive inotropic effects in CA/CPR mice, thus stabilizing or improving circulatory failure, resulting in a better post-resuscitation prognosis.

CA is related to both global and focal I/R injuries of the heart, and one of the main pathological mechanisms of I/R injury is mitochondrial ROS burst. The vicious cycle of mitochondrial destabilization and oxidative stress is a well-known precipitating factor of post-cardiac arrest myocardial stunning [24]. Previously, multiple studies have demonstrated the therapeutic potential of Gn-Rb1 for cardiac I/R injury, and the underlying mechanisms were involved in antioxidant, antiapoptosis, and the regulation of mitochondrial homeostasis [10,34,35,36]. Therefore, we hypothesized that Gn-Rb1 protected against post-CA myocardial stunning by improving oxidative stress and mitochondrial destabilization. We found that Gn-Rb1 reduced the deposition of CA/CPR-induced superoxide and peroxide byproducts such as 4-HNE and NT (Figure 3a–f). In addition, the changes in the antioxidant proteins SOD_2_ and oxidative markers gp91 were a partial remission by Gn-Rb1 (Figure 3g–h). Gp91^phox^ is the catalytic subunit of NADPH oxidase that triggers superoxide anions, and the superoxide contains the maximum burden of free radicals in I/R insult [37]. Superoxide dismutases (SOD), especially the manganese SOD (MnSOD, SOD2), is a mitochondrial antioxidant enzyme that is involved in the scavenger of superoxide [38]. NADH dehydrogenase is a key site to drive ROS production. Jiang et al. [10] reported that the inhibition of mitochondrial NADH dehydrogenase may elucidate the probable mechanism of Gn-Rb1 in alleviating cardiac I/R injury. As such, we examined the NADH dehydrogenase activity and the related subunit protein expression. Notably, Gn-Rb1 reduced the activity of NADH dehydrogenase, and some subunits showed corresponding changes as well, in the context of CA/CPR (Figure 3i,k). However, the expression of the subunits of NADH dehydrogenase may not necessarily be related to the activity of NADH dehydrogenase. In addition, the level of NADH dehydrogenase activity has complex concerns for the oxidation status post-CA and depends on the degree of mitochondrial electron transport chain dysfunction and organ-specificity. Therefore, further study is needed to explore these issues. All in all, our results supported the antioxidant activity of Gn-Rb1 in the CA/CPR context, which is consistent with the experimental results described in the literature [39,40].

Much of the studies in the literature have observed mitochondrial destabilization in the heart after CA/CPR, including morphologic alterations and dysfunctional disorder [41,42,43]. Actually, excessive oxidative stress triggers mitochondrial destabilization and then affects respiratory chain, ATP generation, and cell fate; while mitochondrial destabilization exacerbates oxidative injury in turn. The vicious cycle contributes to myocardial dysfunction. We found that CA/CPR triggered mitochondrial fission, while changes in mitochondrial fusion protein were not obvious (Figure 4d,e). This result was consistent with prior studies that argued for mitochondrial fission as the pathogenesis for post-CA myocardial stunning [44,45]. In addition, a reduction in ATP production and mitochondrial membrane potential (Δψ) is hallmark of mitochondrial defects. However, Gn-Rb1 treatment improves those pathological status. Gn-Rb1 has previously been reported to regulate energy metabolism in other disease settings, such as diabetic cardiomyopathy, heart failure, and I/R injury [10,19,20,21,34]. Here, we demonstrated the regulatory action of Gn-Rb1 on energy metabolism in the CA/CPR heart.

Neurological damage is one of the major cause of disability and death after CA/CPR. Notably, multiple studies have demonstrated the efficacy of Gn-Rb1 in the treatment of cerebral ischemia–reperfusion injury [46,47,48]. However, whether Gn-Rb1 plays a role in CA/CPR-induced cerebral outcomes is yet to be determined. We found that Gn-Rb1 improved the neurological deficit scores in the mouse model of CA/CPR, and the pharmacological effects involved multiple mechanisms such as oxidative stress and apoptosis (Figure 8). The activation of the Keap1/Nrf2 axis might explain, at least in part, the beneficial effects of Gn-Rb1 on post-CA myocardial stunning. However, whether Gn-Rb1 functions similarly in the brain as is proposed in the heart remains unknown. Huang et al. [49] found that Gn-Rb1 had the effects against cerebral I/R injury, which were related to the antioxidative stress and Nrf2/HO-1 signaling pathway. Li et al. [50] suggested that Gn-Rb1 was capable of alleviating cerebral I/R injury in mice by the NF-κB pathway, oxidative stress pathway, and cytokine network pathway. As such, the Nrf2 signaling pathway may also explain the pharmacological effects of Gn-Rb1 on neurological damage following CA/CPR, but further study is required to unveil this issue.

Nrf2, a master transcriptional regulator of redox regulation, activates adaptive responses against oxidative stress, autophagy, apoptosis, and inflammation, through the transcriptional induction of over 600 antioxidant enzymes [51]. Unstimulated, Nrf2 is sequestered by Keap1, and ubiquitinated and degraded in the cytoplasm [52]. Keap1 is inactivated under oxidative stress, allowing Nrf2 to be released from Keap1 and translocated into the nucleus. The activation of the Nrf2 signaling pathway is a major mechanism in the cellular defense against CA/CPR-induced myocardial stunning [53,54]. Our study has several limitations. First, CA was induced in healthy mice with no underlying coronary lesions or cardiac arrhythmia, which may have minimized its clinical relevance. In addition, Gn-Rb1 may exert different effects on cardiac arrest induced by structural heart disease and non-structural heart disease, and it may involve different mechanisms [55,56]. Second, the prognosis of CA was affected by systemic I/R injury, rather than single components. As such, the target organ, possibly Gn-Rb1-primary-specific, remains to be identified. Third, a well-established in vitro model mimicking CA/CPR situations is lacking currently, which disturbs the research of the underlying molecular mechanism. Fourth, we did not test the dose–response study on the mouse CA/CPR model. Fifth, whether Gn-Rb1 functions similarly in the brain as is proposed in the heart remains to be investigated in future. Thus, further investigations are needed to answer these questions.

## 4. Materials and Methods

### 4.1. Reagents and Antibodies

Gn-Rb1 was purchased from Shanghai Yuanye Bio-Technology Co., Ltd. (Shanghai, China). Antibodies against gp91^phox^, SOD_2_, 3-nitrotyrosine, 4 hydroxynonenal, DRP1, DRP1 (phospho S637), DRP1 (phospho S616), Mitofusin 2, OPA1, Fis1, Histone H3, caspases-3, cleaved caspases-3, and VDAC1 were obtained from Abcam (Cambridge, MA, USA). GAPDH, Bax, and Bcl-2 were obtained from Cell Signaling Technology (Beverly, MA, USA). Nrf2, keap1, NQO1, HO1, Ndufs1, Ndufv1, Ndufs6, Ndufs4, Ndufv2, and Ndufa12 were obtained from Abmart (Shanghai, China). Dihydroethidium (DHE) wasobtained from Beyotime (Jiangsu, China).

### 4.2. Experimental Animals

Animal procedures were approved by the Institute’s Animal Ethics Committee of Shanghai Chest Hospital, Shanghai Jiao Tong University (Shanghai, China) (KS(Y)1839). Male C57 mice (8 weeks old) were obtained from Beijing Sibefu Biotechnology Co., Ltd. (Beijing, China) and housed at 25 ± 2 °C with 40–60% humidity under a normal 12 h light/dark cycle, with food and water available ad libitum. As described previously, the CA/CPR model was developed [53]. Briefly, mice were anesthetized using isoflurane (1.5% isoflurane/medical air mixture), and then intubated with a rodent respirator. The right jugular vein was cannulated with a polyethylene tube for fluid administration. ECG monitoring was obtained using limb electrodes. By injecting 0.08 mg of KCl/g body weight, CA was induced, and after an EKG was confirmed to be flat, the ventilator was turned off. Fourteen and a half minutes after onset of CA, mechanical ventilation resumed, and chest compression were delivered at a rate of 350–400 bpm for 15 min. After 20 s of chest compression, mice received 0.4 μg epinephrine/g body weight combined with Gn-Rb1 (50 mg/kg), or epinephrine only. This dose of Gn-Rb1 was given on the basis of previously published reports [10,17]. Additional doses of epinephrine were given at 1 min intervals until return of spontaneous circulation (ROSC) or after 5 min of CPR (Figure 1a). A total of 3 h after ROSC, mice were euthanized. Randomization was performed using simple randomization method via a random number table. The left ventricular apex tissues were embedded in OCT compound or kept in paraffin, and the residual heart tissues were then frozen at −80 °C for molecular analysis.

### 4.3. Echocardiography

Cardiac structure and function were determined using the Vevo770 system (VisualSonics, Toronto, Canada) at the indicated times of post-ROSC. The mice were anaesthetized by inhalation of 2% isoflurane, and M-mode images of the parasternal long axis were obtained to calculate left ventricular fractional shortening (LVFS), cardiac output (CO), and left ventricular ejection fraction (LVEF), as previously described [53].

### 4.4. Immunohistochemistry

Serial sections (5 µm) were prepared from formalin-fixed, paraffin-embedded left ventricular apex tissues. The 4 hydroxynonenal (4HNE) staining and 3-nitrotyrosine (NT) staining were used to evaluate the oxidative stress in myocardium. Quantitative image analysis of immunohistochemistry was performed using Image J analysis software [38].

### 4.5. Dihydroethidium (DHE) Staining

The collected left ventricular apex tissues and brain tissues were embedded in OCT and cut into 6 μm sections. Cryosections were washed 3 times for 5 min using PBS and incubated with 10 μmol/L dihydroethidium for 30 min. After washing with PBS 3 times again, the slides were viewed under a fluorescence microscope (DM2500, Leica). The maximum excitation wavelength is 300 nm, and the maximum emission wavelength is 610 nm. The fluorescence intensity of DHE staining was measured using ImageJ software (version 2.0.0).

### 4.6. Transmission Electron Microscopy (TEM)

Mitochondrial morphology was evaluated by transmission electron microscopy, as previously described [53]. Briefly, the left ventricles were fixed, dehydrated, embedded, and cut into ultrathin slices (70 nm), and then observed and imaged using TEM (Hitachi HT-7800, Tokyo, Japan).

### 4.7. Western Blot Analysis

Extraction of cytoplasm and nuclear proteins was realized using Nuclear and Cytoplasmic Protein Extraction Kit (Beyotime, China). Total proteins were extracted from heart tissues, brain tissues, or primary cardiomyocytes according to product manual (Roche, USA). Protein concentration was quantified using a BCA protein assay (Thermo Fisher Scientific). The amount of protein was adjusted to 20 μg per lane. Proteins were separated using 7.5–12.5% SDS-PAGE and transferred onto 0.22 μm PVDF membranes. After being blocked with 5% BSA for 1 h and rinsed with PBS, the membrane was incubated for 12 h at 4 °C with the primary antibody. On the following day, the primary antibodies were removed with three rinses of PBS. The immunoblot bands were visualized using chemiluminescence (Millipore) via ImageQuant LAS 4000 Imager (General Electric, Pittsburgh, PA, USA.) after incubation with the corresponding secondary antibodies (ab288151, 1:10000, Abcam, Cambridge, UK) for 1 h at room temperature. The ratio of the gray value of the target bands to the internal reference band (GAPDH) was used as the relative expression of the protein.

### 4.8. Real-Time Quantitative PCR

Total RNA from cells or tissues was extracted using TRIzol^®^ reagent (Invitrogen, USA). Isolated RNA was reverse-transcribed and duplicated using PrimeScript™ RT Master Mix (Vazyme, Nanjing, China) and SYBR qPCR master mix (Vazyme, Nanjing, China) in iScript cDNA Synthesis Kit (Takara BIO, Otsu, Japan) and the Light-Cycler 480 Real-Time PCR System (Roche, San Francisco, CA, USA). Primers sequences are listed in Supplementary Table S1. The results were normalized to GAPDH and expressed as percentage of controls.

### 4.9. Terminal Deoxynucleotidyl Transferase dUTP Nick-End Labelling (TUNEL) Assay

TUNEL staining was performed using a TUNEL Apoptosis Assay Kit (Beyotime, Shanghai, China). TUNEL-positive nuclei were identified as apoptotic cells stained with FITC (green), and nuclei were simultaneously counterstained with DAPI. Images were captured using a Leica DMIRE2 fluorescence microscope. The excitation wavelength range was 450–500 nm, and the emission wavelength range was 515–565 nm. TUNEL-positive signals were normalized to the total nuclei signals for each field.

### 4.10. Mitochondrial Isolation

Mitochondrial isolation from the hearts or NRCMs was performed using the Mitochondrial Isolation Kit (Beyotime, Shanghai, China) [25]. Briefly, cells and tissue were mechanically homogenized for 30 strokes using a tight pestle on ice in mitochondrial isolation buffer added with PMSF, and centrifuged at 600 g for 10 min at 4 °C, and then the resulting supernatant was centrifuged again at 11,000 g for 10 min at 4 °C to obtain mitochondria. The isolated mitochondria and cytoplasm were used for subsequent experiments.

### 4.11. Cell Culture and Transfection

The neonatal rat cardiomyocytes (NRCMs) were isolated from heart ventricles of 1- to 3-day-old SD rats [57]. In brief, the ventricles were minced and digested with collagenase type II (Invitrogen) and pancreatase myocyte digestion buffer (Sigma-Aldrich, USA). After differential adhesion, the supernatants of primary cultures of myocardial cells were plated and then grown in DMEM with 10% FBS (fetal bovine serum, GIBCO, Billings, MT, USA), 100 U/mL penicillin, and 100 μg/mL streptomycin at 37 °C and 5% CO_2_ for 48 h. NRCMs at a density of 50–70% were transfected with Nrf2 small interfering RNAs (siRNAs, 50 nM, purchased from Genepharma, Shanghai, China) to silence Nrf2 using Lipo3000 (Invitrogen, Carlsbad, CA, USA). Sequences of the Nrf2 siRNA sequence were as follows: forward oligo, 5′-GGAUGAAGAGACCGGAGAAUU tt-3′, reverse oligo, and 5′-AAUUCUCCGGUCUCUUCAUCC tt-3′. We investigated the following groups after 72 h of transfection. Standard incubators were used to culture the blank group without transfection. Cells transfected with Nrf2 siRNA (Nrf2 siRNA group), or negative control siRNA (NC group) were exposed to hypoxia for 12 h and reoxygenation for 3 h, to simulate I/R injury. Based on Nrf2 siRNA group and NC group, Nrf2 siRNA+Rb1 group and NC + Rb1 group were treated with Gn-Rb1 (10 µM) during reoxygenation for 3 h [10,14,34].

### 4.12. Detection of Cellular Reactive Oxygen Species

Production of reactive oxygen species (ROS) was detected using DCFH-DA fluorescent probe kit (Beyotime, Shanghai, China). In brief, the plates were incubated in the dark for 20 min with DCFH-DA (10 M), and then cells were washed 3 times to remove DCFH-DA. Subsequently, cells were visualized using Leica DMIRE2 fluorescence microscope. We used 488nm excitation wavelength and 525 nm emission wavelength.

### 4.13. Detection of Mitochondrial ROS (mROS)

mROS production was measured using MitoSOX ^Red^ (Invitrogen, USA). In brief, cells from different groups were washed 3 times with PBS and incubated for 30 min with 1 µM MitoSOX ^Red^, and then counterstained with Hoechst. After washing 3 times with PBS, images of mROS level were obtained (Excitation/Emission 396/610 nm) using a fluorescence microscope (Leica DM400B, Leica Microsystems, Ltd., Wetzlar, Germany).

### 4.14. Assessment of Neurological Function

A 12-point mouse neurologic scoring system was used to assess neurological deficits in mice after CA [58]. Six domains were evaluated with scores ranging from 0 (no response) to 2 (normal): righting reflex, motor-focal, breathing, spontaneous movement, paw pinch, and motor-global. For each domain, a blinded score was calculated and summed to obtain a neurologic score. The comparisons between the two groups were performed using unpaired *t*-test.

### 4.15. Cellular ATP Assay

Cellular ATP levels were measured using the Enhanced ATP Assay Kit (Beyotime, Shanghai, China). The cardiac tissues and treated NRCMs were lysed with ATP assay lysis buffer. The lysed cells were centrifuged in 12,000 g for 5 min at 4 °C, and then we collected supernatant. Afterward, we added ATP detection working solution to a 96-well black plate and incubated for 5 min, and then added supernatant to the plate quickly. The RLU of samples was detected by luminometer within 30 min. We normalized the luminescence signals to the protein concentrations in order to calculate the total ATP levels.

### 4.16. Mitochondrial Membrane Potential Examination

A mitochondrial membrane potential assay kit with JC-1 (Beyotime, China) was used to detect the mitochondrial membrane potential. Briefly, cells were incubated with JC-1 working solution for 20 min, and then washed twice with JC-1 staining buffer. Cells were imaged using fluorescence microscope (Leica Microsystems). The potential gradient of the mitochondrial membrane potential (Δψm) was indicated by the ratio of green fluorescence to red fluorescence. For heart tissue, the isolated mitochondria were incubated with JC-1, as described previously. Green (488 nm excitation and 530 nm emission) and red (543 nm excitation and 590 nm emission) fluorescence were detected by Fluoromax-2 spectrophotometer (Horiba Jobin Yvon, Paris, France).

### 4.17. NADH Dehydrogenase Activity Assays

NADH dehydrogenase activity assay was performed using the kit of Tong Wei (TW, reagent, Shanghai, China) according to the manufacturer’s instructions. Briefly, mash the tissue with an appropriate amount of normal saline, and then put it in 3000 centrifuge for 10 min to obtain the supernatant. In the micropores coated with NADH dehydrogenase antibodies, samples, standard samples, and HRP-labeled antibodies were added successively, incubated (37 ℃, 60 min), and washed thoroughly. The substrate TMB was used to develop color. The absorbance (OD value) was determined by enzyme-labeling instrument at 450 nm wavelength, and the activity of the sample was calculated. Protein concentration was determined by BCA method.

### 4.18. Statistical Analyses

The normality of data distribution was tested using the Shapiro–Wilk normality test. Normally distributed variables were presented as means ± standard deviation (SD), while categorical variables were presented as frequencies or percentages. To test for statistical significance, continuous variables following normal distribution were compared using Student’s *t*-test, while data that did not follow a normal distribution were analyzed by using non-parametric test. Comparisons among multiple groups were performed using one-way ANOVA. Categorical data were compared using the Chi-square test. For survival analysis, Kaplan–Meier survival analysis was used, and comparisons between groups were made using a log-rank (Mantel–Cox) test. *P* < 0.05 was considered statistically significant. All analyses were performed using Graph-Pad Prism 8 (GraphPad Software, LLC, San Diego, CA, USA).

## 5. Conclusions

To sum up, our study provides the first evidence that Gn-Rb1 protects against post-cardiac arrest myocardial stunning, partly via alleviating oxidative stress and mitochondrial destabilization through the activation of the Keap1/Nrf2 signaling pathway, which sheds insight into the role of Gn-Rb1 as a new prospective agent against CA. Further studies, in particular clinical trials, will be important to confirm its therapeutic value in a clinical setting.

## Data Availability

The raw data supporting the conclusion of this article will be made available by the authors, without undue reservation.

## Supplementary Materials

The following supporting information can be downloaded at: https://www.mdpi.com/article/10.3390/ijms24055059/s1.
