# Supplementary material for: Ginsenoside Rb1 Improves Post-Cardiac Arrest Myocardial Stunning and Cerebral Outcomes by Regulating the Keap1/Nrf2 Pathway
Source: Int J Mol Sci. 2023 Mar 6;24(5):5059. doi: 10.3390/ijms24055059 (PMC10003120; doi:10.3390/ijms24055059)
Supplement: Supplementary file 1 [file ijms-24-05059-s001.zip › Table S1.pdf]

Supplemental Table S1. Primers for PCR	
NPCR production	Oligoes for mutations (forward/reverse)
Mouse Nrf2	CCATGTGTGGCAGTCCATGAT
	GCAGGCATACCATTGTGGAT
Rat Nrf2	GGTTGCCCACATTCCCAAAC
	CAGGGCAAGCGACTGAAATG
